# Predictors of treatment outcomes for patients with persistent physical symptoms in primary care: findings from a cluster randomised controlled trial

**DOI:** 10.3399/BJGPO.2024.0004

**Published:** 2024-12-11

**Authors:** Cathrine Abrahamsen, Knut Reidar Wangen, Morten Lindbaek, Erik Lønnmark Werner

**Affiliations:** 1 Department of General Practice, Faculty of Medicine, University of Oslo, Oslo, Norway; 2 Department of Health Management and Health Economics, University of Oslo, Oslo, Norway

**Keywords:** pain, medically unexplained symptoms, somatisation, primary health care

## Abstract

**Background:**

Persistent physical symptoms (PPS) are consistently prevalent among primary care patients. PPS can negatively affect quality of life, healthcare costs, and work participation. In a previous study, we found substantially improved outcomes and reduced sick leave for patients treated by a work-focused communication tool, known as the Individual Challenge Inventory Tool (ICIT), compared with a control group.

**Aim:**

To examine predictors of treatment outcome in patients who received treatment by ICIT, delivered by the patient’s GP.

**Design & setting:**

This study is based on the findings of our previous cluster randomised controlled trial undertaken in Norway.

**Method:**

Regression analyses of the intervention group were used to identify predictors (all measured at baseline) of improvements in Patient Global Impression of Change (PGIC) and sick leave after 11 weeks follow-up.

**Results:**

Living alone predicted improvement in the adjusted model (odds ratio [OR] 4.03, 95% confidence interval [CI] = 1.33 to 12.25, *P* = 0.014). Receiving long-term benefits predicted improved PGIC in both the unadjusted (OR 2.30, 95% CI = 1.21 to 4.39, *P* = 0.011) and adjusted models (OR 2.46, 95% CI = 1.04 to 5.83, *P* = 0.040). In addition, living alone predicted reduced sick leave in the adjusted model (OR 3.23, 95%CI = 1.11 to 9.42, *P* = 0.032).

**Conclusion:**

In general, there were few factors to predict the outcome of the work-focused communication tool. We therefore suggest that this work-focused communication tool is applicable to most patients with PPS. GPs may consider using the ICIT for all primary care patients who exhibit ineffective coping strategies in their daily lives and work, especially those who live alone.

## How this fits in

Cognitive behavioural therapy (CBT) is effective in specialised settings for treating patients with persistent physical symptoms (PPS), but its efficacy in primary care is limited. Using a structured communication tool (Individual Challenge Inventory Tool [ICIT]) in primary care for patients with PPS has shown improvements in function, symptoms, quality of life, and reduced sick leave. This study suggests that the structured communication tool can benefit GPs in treating patients with PPS, regardless of sex, age, education, or depressive symptoms. Clinical implications include recommending the structured communication tool for primary care patients with unproductive coping behaviours, particularly those who live alone.

## Introduction

Patients with medically unexplained physical symptoms experience symptoms and functional limitations that cannot be explained through objective investigations or pathology. Patients with symptoms turn to their GPs for assistance. Typically, these episodes are brief and tend to improve over time, leading to favourable outcomes. However, challenges arise when the symptoms persist for a significant duration and become severe in nature.^1^ Recently, researchers have argued for the term persistent physical symptoms (PPS) to be used. This is because the term is better accepted by patients and the underlying mechanisms for the condition, to a large extent, have been revealed.^2^ In this study we will therefore use the term PPS. While the overall prevalence of patients with PPS in primary care is estimated to be around 40%,^3^ the number of patients with severe PPS in general practice is reported to be 16%.^1^ Patients with PPS frequently suffer from psychological distress, social isolation, and reduced quality of life. This commonly leads to a dependency on healthcare services, resulting in amplified healthcare utilisation rates and expenses connected to sick leave.^4–6^

CBT is often used to treat PPS, but its effectiveness is only moderate, especially in primary care.^7,8^ Previous interventions in primary care have not reduced sick leave in patients with PPS.^9^ CBT interventions for similar patient groups have struggled to prove their effectiveness in helping patients return to work.^10^ Lagerveld *et al* emphasise the importance of including a focus on returning to work in CBT interventions.^11^ To address the need for an intervention to assist GPs in managing patients with PPS and assessing sick leave, a work-focused communication tool (ICIT) using a cognitive-behavioural approach was developed and found feasible in primary care in Norway.^12^

We have previously reported from a cluster randomised controlled trial (cRCT) performed in primary care in Norway, where we compared the effectiveness of this work-focused structured communication tool to usual care in patients with PPS. In contrast to the usual care, the intervention group reported significant improvement in function, symptoms, quality of life, and decreased sick leave.^13^

Sarter *et a*l found that increased symptom load and reduced functional capacity in patients with PPS before therapy were associated with less favourable treatment outcomes of CBT.^14^ Previous studies of clinical characteristics of patients in relation to therapy outcomes has proven insightful for patients with depression and pain.^15–17^ Pain in multiple sites, high pain severity, older age, baseline disability, and longer pain duration have been identified as potential predictors of disability.^18^ However, in the case of patients with subacute pain, there was limited evidence to support the association between anxiety, depression, and disability. These findings suggest that the role of anxiety and depression in contributing to disability in pain conditions may not be as significant as previously assumed.^19^

In our previous study, we found a decrease of 27% in sick leave among patients with PPS following the intervention with the ICIT, and 76% of the patients reported a significant overall improvement in function, symptoms, and quality of life.^13^ In this study, we aimed to identify characteristics of those patients who achieved these improvements. Such associations could help clinicians to identify and tailor their use of the structured communication tool at the encounter.

Our objective was to assess whether the predictors had any impact on the treatment outcome or if they indicated a potential for a negative outcome. Drawing from previous research, our hypothesis was that patients with depression and those manifesting multiple symptoms would face an elevated risk of experiencing an unfavourable treatment outcome.

## Method

### Design and setting

This study extends the findings of our previous cRCT. Details about randomisation, inclusion and exclusion criteria, comparison of the intervention and usual care group before the first consultation, and how the study was conducted are reported elsewhere.^13^ In this study, the analysis specifically focuses on the intervention group. The baseline questionnaires were completed by 231 participants in the intervention group. The follow-up form was completed for 223 participants (97%). Eighty-four per cent of the participants were female and there was an average age of 45 years (standard deviation = 13.2). Finally, more than half (58%) of the participants reported experiencing feelings of depression within the past week.^13^

**Figure 1.**
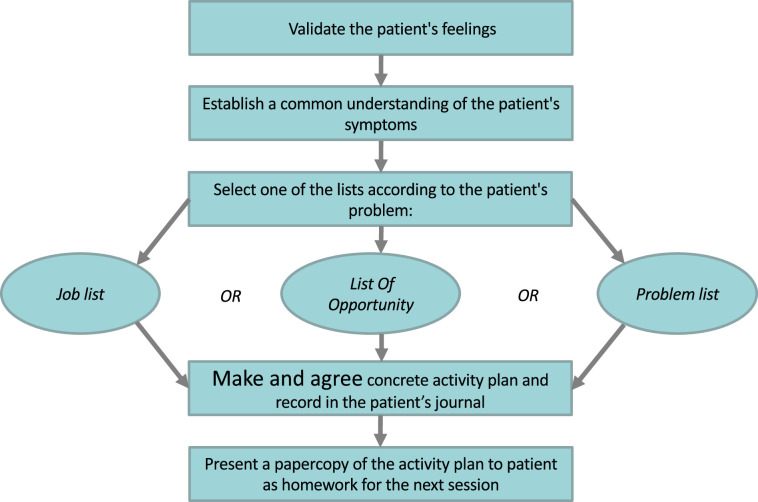
Individual Challenge Inventory Tool (ICIT): a structured communication tool with a cognitive work approach

The intervention was delivered by the participant's GP during a minimum of two consultations, utilising the structured communication tool. This tool, ICIT, was developed by the first author, and is grounded in CBT principles. The ICIT is a structured communication tool with a cognitive work approach that helps patients improve self-care and assists doctors in evaluating sick leave. The ICIT guides discussions from greetings to final remarks, promoting patient coping and mutual understanding of PPS causes and impacts. The structured communication tool follows steps to maintain conversation structure, validate emotions, understand patient concerns, and create specific action plans based on work or personal challenges. Figure 1 depicts the structure of the ICIT. Detailed information about the ICIT and a comprehensive description of its knowledge base for structured communication can be found in another resource.^13^

Since 2001, Norway has a GP scheme that offers every citizen the right to sign up at a specific general practice. Each consultation typically lasts 15–20 minutes but can be extended at the GP’s discretion. Furthermore, Norwegian GPs combine the roles as health provider and issuing any sick leave. The worker may be sick listed with 100% compensation for up to 1 year, at the GP’s discretion. Hence, the GP’s role in sickness absence is substantial.

Each participant was mandated to complete a series of questionnaires both before their initial GP session and following their last session in the 11-week study. These questionnaires assessed the following various factors that we considered as potential predictors of treatment outcomes:

- baseline participant characteristics, including age, sex, educational level, living alone, and the number of children;
- employment status;
- sick leave;
- receipt of long-term benefits;
- depression (all participants were required to respond to the following question before their initial and final consultations in the study: *'During the past 14 days, have you experienced feelings of sadness or depression*?' They were then given five possible choices for response, ranging from *'fits very well'* to *'fits very poorly'*. Previous research has demonstrated the reliability and validity of utilising a single question to gauge potential depression in patients);^20^ and
- the number of symptoms considered as PPS documented by the participant's GP.

### Measures

The primary outcome variable utilised in our predictive analyses was the Patient Global Impression of Change (PGIC), evaluated during the 11-week follow-up, in line with our prior study.^13^ The PGIC evaluates alterations in clinical status based on participants self-reported perceptions of changes in their overall functioning, symptoms, and quality of life from baseline to follow-up. Participants were presented with a single query: *'Please describe the changes in your functioning, symptoms, and overall quality of life since receiving treatment from your GP.'* They were then provided with seven response options, spanning from *'very much better'* to *'very much worse'*. The PGIC is notable for its concise yet patient-centred approach, enabling it to encompass subjective improvements as well as potential adverse events. It has established validity in chronic pain trials, as affirmed by prior research.^21^

To facilitate our analysis we adopted dichotomous variables as follows: participants who reported improvement selected one of the three options from *'minimally improved'* to *'very much better'*, compared with those reporting no effect who chose one of the four alternatives from *'no change'* to *'very much worse'*.

The secondary outcome was to evaluate how the predictors mentioned earlier could influence sick leave. At 11 weeks, the intervention group had a 27-percentage point decrease in sick leave, compared with a 4-percentage point decrease in the usual care group.

We determined that a 10% reduction in sick leave was of clinical significance. As a result, we created a dichotomous variable, distinguishing between participants who experienced a ≥10% reduction in sick leave at the 11-week follow-up and those who did not achieve 10% reduction in sick leave.

During the study, the GPs documented the participants' sick leave status at the following three critical time points: baseline; during the 11-week follow-up; and after the final session of the study. At each assessment, participants were classified as being on full-time sick leave (yes or no) or on partial sick leave, which was quantified as a percentage. More information can be found elsewhere.^13^

### Statistical analyses

Data were analysed using Stata (version 17.0). We separately estimated logistic regression models for PGIC improvement and sick leave improvement. We considered both continuous and dichotomous factors as predictor variables, including sex, living situation, employment status, sick leave, and long-term benefits. In addition, age and depression were analysed as continuous predictors. Unadjusted models were estimated with one predictor only. Adjusted models, which included all predictor variables from the unadjusted models, were conducted to determine the strength of the associations between the predictors and the outcomes. In each unadjusted model, we used all non-missing observations for that predictor. In the adjusted models, we used complete cases only.

## Results

### Main findings

An analysis of baseline participant characteristics revealed that receiving long-term benefits was the only significant predictor associated with a positive outcome after an 11-week follow-up (Table 1). However, after implementing a fully adjusted model, we found that living alone (odds ratio [OR] 4.03, 95% confidence interval [CI] = 1.33 to 12.25, *P* = 0.014) and receiving long-term benefits (OR 2.46, 95% CI = 1.04 to 5.83, *P* = 0.040) had a significant impact on achieving a favourable outcome. Other predictors, such as age, sex, educational level, number of children, employment status, sick leave, depression, and the number of persistent physical symptoms, did not have a significant impact on the outcome.

**Table 1.** Odds ratios with confidence intervals obtained from unadjusted and adjusted logistic models for PGIC improvement

	Unadjusted models, *n* = 223^a^			Adjusted model, *n* = 154		
**Characteristic**	**OR**	***P***	**95% CI**	**OR**	***P***	**95% CI**
Depression	1.05	0.732	0.79 to 1.39	1.06	0.756	0.74 to 1.51
Female	1.41	0.403	0.63 to 3.19	1.68	0.348	0.57 to 5.00
Age	0.99	0.514	0.97 to 1.02	0.99	0.437	0.95 to 1.02
Number of children	1.03	0.864	0.75 to 1.41	1.29	0.244	0.84 to 1.99
Educational level						
High school (10–13 years)	0.98	0.968	0.37 to 2.60	1.46	0.554	0.42 to 5.05
Higher education institutions (>13 years)	1.33	0.567	0.50 to 3.57	1.94	0.299	0.55 to 6.83
Living alone	1.65	0.161	0.82 to 3.33	4.03	0.014	1.33 to 12.25
Long-term benefits (>12 months)	2.30	0.011	1.21 to 4.39	2.46	0.040	1.04 to 5.83
Full-time sick leave	1.57	0.271	0.70 to 3.48	1.72	0.379	0.51 to 5.73
Number of PPS symptoms	0.87	0.265	0.67 to 1.12	1.11	0.570	0.78 to 1.57

^a^Owing to missing values, the number of observations varied between 173 and 223 in the unadjusted models. The adjusted model included all predictor variables from the unadjusted models. OR = odds ratio. PGIC = Patient Global Impression of Change. PPS = persistent physical symptoms.

### Additional findings

The second objective of this study was to determine any associations between the predictors and sick leave. Results from the unadjusted analysis revealed that none of the predictors were associated with a decrease in sick leave (Table 2). However, after implementing a fully adjusted model, the analysis suggests that living alone was significantly linked to reduced sick leave (OR 3.23, 95% CI = 1.11 to 9.42, *P* = 0.032). In contrast, other predictors, including age, sex, educational level, number of children, employment status, receipt of long-term benefits, depression, and the number of medically unexplained physical symptoms, did not predict any decrease in sick leave.

**Table 2.** Odds ratios with confidence intervals obtained from unadjusted and adjusted logistic models for sick leave improvement

	Unadjusted models, *n* = 158^a^			Adjusted model, *n* = 104		
**Characteristic**	**OR**	***P***	**95% CI**	**OR**	***P***	**95% CI**
Depression	1.10	0.512	0.82 to 1.47	1.26	0.231	0.86 to 1.86
Female	0.97	0.946	0.41 to 2.31	1.08	0.899	0.31 to 3.76
Age	1.02	0.296	0.99 to 1.05	0.99	0.628	0.95 to 1.03
Number of children	1.31	0.106	0.94 to 1.81	1.41	0.150	0.88 to 2.24
Educational level						
High school (10–13 years)	1.33	0.615	0.43 to 4.09	0.96	0.961	0.22 to 4.16
Higher education institutions (>13 years)	1.23	0.709	0.42 to 3.59	1.72	0.441	0.43 to 6.80
Living alone	1.94	0.065	0.96 to 3.91	3.23	0.032	1.11 to 9.42
Long-term benefits (>12 months)	0.97	0.960	0.32 to 2.91	1.38	0.654	0.33 to 5.73
Number of PPS symptoms	1.13	0.384	0.86 to 1.48	1.32	0.158	0.90 to 1.94

^a^Owing to missing values, the number of observations varied between 120 and 158 in the unadjusted models. The adjusted model included all predictor variables from the unadjusted. OR = odds ratio. PPS = persistent physical symptoms.

## Discussion

### Summary

The aim of this study was to explore associations between favourable outcomes in our previous cRCT on patients with PPS and patient characteristics.^13^ These associations can guide GPs in selecting suitable patients for ICIT management. Our results suggest that living alone and receiving long-term benefits could be associated with favourable outcomes. Contrary to our initial hypothesis, patients with depression and multiple symptoms did not experience less favourable treatment outcomes with the ICIT. These findings indicate that the structured communication tool can be beneficial for GPs in treating patients with PPS, irrespective of sex, age, education, and whether the patients feel depressed or not.

### Strengths and limitations

Even though the sample size is a strength of this study, a longer follow-up period of the cRCT would have been preferred.^13^ Previous research found that 14% of patients who had been unemployed for >13 weeks did not go back to work.^22^ Based on this evidence, we believe that the selected 11-week follow-up period was considered clinically significant in evaluating sick leave as an outcome.

It should be noted that finding clear connections between participant characteristics and the efficacy of the conversation tool may be challenging when 76% of participants reported improved functioning, reduced symptoms, and an increased quality of life. Therefore, it may prove to be equally challenging to establish reliable correlations between specific patient traits and treatment outcomes.

Most participants in the intervention group experienced improvements and were recipients of long-term benefits. We believe this is noteworthy because individuals on long-term benefits might feel overlooked or not prioritised within the healthcare system. This study demonstrates that individuals living alone and/or receiving long-term benefits have the potential for positive outcome.^13^ Given better function, reduced symptoms, and better quality of life could reduce unnecessary referrals to secondary care and their associated costs.

### Comparison with existing literature

While previous research has suggested factors such as increased symptom load and impaired function as predictors of unfavourable treatment outcomes for patients with PPS,^14^ our study did not find such associations. We have identified living alone as a significant predictor of decreased sick leave, as well as improved function, symptoms, and quality of life. For individuals who live alone, being actively involved in work may have a greater impact on their daily routines and social interactions. It is possible that patients who live alone have fewer resources to sustain their work involvement owing to a lack of support. However, when their GP utilises work-focused cognitive therapy and collaborates with the patient to develop a written plan on how to preserve their engagement in work despite their ailments, patients experience a decrease in sick leave.^13^ This finding complements with a previous study that has also demonstrated a reduction in sick leave when employing work-focused cognitive therapy as opposed to standard CBT for comparable patient groups.^11^ Addressing employment-related concerns and formulating strategies to support employment can produce favourable results in sick leave outcomes,^13^ especially for individuals who live alone.

Pain in multiple sites, high pain severity, older age, baseline disability, and longer pain duration have been identified as potential predictors of disability.^18^ In the present study we were unable to find such associations. Previous studies with similar patients found limited evidence to support the association between anxiety, depression, and disability. These findings suggest that the role of anxiety and depression in contributing to disability in pain conditions may not be as significant as previously assumed.^19^ This is in line with our findings, and we found it particularly interesting that being depressed did not predict better outcomes. While patients with PPS often present with psychological comorbidities, such as anxiety and depression, it is worth noting that PPS is not a psychiatric diagnosis,^23^ and it may be of importance to the patient to make such a distinction.^24^ Consequently, the presence of depression and having multiple symptoms should not deter GPs from using the ICIT to structure communication.

### Implications for practice

In this study, we found that living alone seemed to predict improvements in function, symptoms, quality of life, and reduced sick leave for patients who are treated using the structured communication tool, ICIT. In our opinion, it is likely that this is valid for most patients with PPS. Moreover, we find it equally likely that the structured communication tool will be successful in consultations where general function, symptom load, reduced sick leave, and quality of life are crucial factors for improvement. Clinical implications include recommending the ICIT for primary care patients with unproductive coping behaviours, especially those who live alone.

## Data

An anonymised dataset will be provided on reasonable request, pending necessary approvals from relevant authorities.
